# A synthetic population for agent-based modelling in Canada

**DOI:** 10.1038/s41597-023-02030-4

**Published:** 2023-03-21

**Authors:** Manon Prédhumeau, Ed Manley

**Affiliations:** University of Leeds, School of Geography, Leeds, LS2 9JT UK

**Keywords:** Scientific data, Socioeconomic scenarios

## Abstract

In order to anticipate the impact of local public policies, a synthetic population reflecting the characteristics of the local population provides a valuable test bed. While synthetic population datasets are now available for several countries, there is no open-source synthetic population for Canada. We propose an open-source synthetic population of individuals and households at a fine geographical level for Canada for the years 2021, 2023 and 2030. Based on 2016 census data and population projections, the synthetic individuals have detailed socio-demographic attributes, including age, sex, income, education level, employment status and geographic locations, and are related into households. A comparison of the 2021 synthetic population with 2021 census data over various geographical areas validates the reliability of the synthetic dataset. Users can extract populations from the dataset for specific zones, to explore ‘what if’ scenarios on present and future populations. They can extend the dataset using local survey data to add new characteristics to individuals. Users can also run the code to generate populations for years up to 2042.

## Background & Summary

The trajectory of spatial and transportation modelling is undoubtedly towards more granular representations of behaviour. Facilitated by the growth in richer, finer-grained mobility data, increased use of individual-level modelling in transportation planning is widely recognised^1^. The predominant methodology in this area is Agent-Based Modelling (ABM), an approach which involves modelling heterogeneous individual agents who act and interact autonomously. ABM has been recently applied to urban planning^2,3^, future transportation^4–6^, policy evaluation^7–9^ or simulating disease outbreaks and interventions^10,11^. Frameworks for developing ABMs, such as MATSim^12^ and AIMSUN^13^ for transportation, and Repast and NetLogo, have support these applications.

When applied to real-world cases, ABM can benefit from using realistic synthetic populations of agents^14^. A realistic synthetic population does not attempt to represent every real individual as an agent. But to qualify as realistic, the synthetic population must be composed of agents that have socio-demographic attributes that could be found in a real individual, with statistical distribution of characteristics similar to those of the real population. If the synthetic population involves relations between agents, such as household formation or a spatial dimension, the population must also have realistic statistical characteristics at these levels. The synthetic population can then act as a test bed to evaluate the impact of public policies or to conduct experiments that would be costly, unethical, or infeasible with real population data.

With these purposes in mind, several works proposed open-source synthetic populations; for the UK^15^, the US^16,17^, or often for more specific geographic areas like for the Ile-de-France region^18^ (France), Tallinn^19^ (Estonia), American Samoa^20^ (US), California^21^ (US) or Australian capital cities^22^. Similarly, several works have produced synthetic populations for Canadian cities. A synthetic population has been developed for Halifax in order to simulate individuals’ decisions along their life-course^23^. A geospatial synthetic population has been developed for the island of Montreal in order to analyse the residential location choice of the new immigrant populations^24^. The TASHA (Toronto Area Scheduling Model for Household Agents) model^25^, designed to study individual activity schedules and travel patterns for the Greater Toronto area, includes a synthetic population directly sampled from the Transportation Tomorrow Survey data. However, this travel survey is conducted only in the Greater Golden Horseshoe Area (south-central Ontario) and there is no equivalent at the national level. With a focus on the methodology rather than producing an open synthetic dataset, a synthetic population has been proposed for the Atlantic region for the year 2006^26,27^. Furthermore, the national statistical agency of Canada develops “The Social Policy Simulation Database and Model (SPSD/M)”, a synthetic population dataset that is specifically designed for analysing the tax and transfer policies at the province spatial level (https://www150.statcan.gc.ca/n1/en/catalogue/89F0002X). They also produce Demosim, a model designed to generate population projections at fine-scale level, but the model is not available to external users.

Although several works have developed synthetic populations for some regions of Canada, there is no up-to-date, open-source synthetic population for all of Canada. To overcome this gap, this paper details the creation of an individual-level synthetic population at a fine geographical scale for the all Canada, for the years 2023 and 2030.

A commonly used data source for population synthesis is the population census. Canadian census data is released as aggregated statistics for various levels of geography and two Public Use Microdata Files (PUMFs), one for individuals and one for households. However because it is a complex and extensive process, population censuses are conducted only every five years in Canada. Moreover, the census raw data need to be carefully processed by the national statistical agency to ensure confidentiality and accuracy before census results are released. This means that census data is published progressively between 9 months (for population counts) and 3 years (for households microdata) after the census has taken place, and the data are therefore no longer up to date at the time of their publication. A solution adopted by the SPENSER model^15^ is to synthesise a base population using past UK census data and then project the population to represent the present or the future. The method we used to generate the dataset is inspired by the SPENSER approach, but was adapted to the data available in Canada (no household projections, aggregate population data at a slightly higher level than in the UK, population projections by age and sex available only at the provincial level).

Many methods have been proposed to generate synthetic populations^28^:

- *Synthetic reconstruction* like Iterative Proportional Fitting (IPF)^29^ and Iterative Proportional Updating (IPU)^30^, which combines sample data and aggregate local statistics to compute the weights reflecting each sample individual’s representativeness in the local zone. Müller^31^ proposed a Hierarchical IPF method which sample the hierarchical PUMF to directly generate a synthetic population of households and individuals. However, this method assumes a representative sample of both households and individuals, and the hierarchical PUMF of the 2016 Canadian census contains only 1% of individuals which limits its representativeness regarding individuals.
- *Combinatorial optimisation* with algorithms such as hill climbing^32^ or simulated annealing^33^, which consists in duplicating real individuals from a sample and iteratively updating the synthetic population in order to better fit the real population. While the combinatorial optimisation approach has shown great potential, the optimization algorithms used can get stuck in local optima and have a high computational complexity for large populations. Most applications of combinatorial optimisation have therefore been restricted to small population sizes^26^ and the approach is not suitable for generating a complete Canadian population.
- *Statistical learning* using Markov chain Monte Carlo simulation (MCMC)^34^, Hidden Markov Model (HMM)^35^ or Bayesian network^36,37^, where individuals and attributes are sampled one after another and dependent on previous states, with transitions built from partially known distributions. More recently, deep learning methods have also been proposed^38^, using a variational autoencoder to learn the joint distribution of all individuals in the sample. However, statistical learning methods fail to satisfy the conditional attributes distributions while satisfying the aggregated distributions of all variables simultaneously^28^. In these methods a post-processing step using a synthetic reconstruction method is required to accurately match the observed distributions at a small area level.

Following the decision tree provided by Yameogo *et al*.^28^ to identify the most suitable methods for generating a two-layered synthetic population, we decided to apply the synthetic reconstruction approach. The most common synthetic reconstruction approach is synthetic reconstruction with IPF^29^. This method uses sample data as a seed and assigns each individual in the population sample a weight such that the weighted population shows predefined marginal distributions for attributes aggregated at a small area level. IPF has the advantage to be fast, simple and deterministic^39^, but generates fractional weights instead of integer populations, which is an important limitation when the synthetic population is to be used in an ABM with a integer number of agents. A comparison of integerisation procedures^40^ showed that the ‘truncate, replicate, sample’ and ‘proportional probabilities’ methods were more accurate than the ‘simple rounding’, ‘inclusion threshold’ or ‘counter-weight’ methods. However, the integerisation process can still introduce a mismatch between the original and simulated marginal distribution^40^. To overcome this issue, a probabilistic resampling method called Quasirandom Integer Sampling (QIS)^41^ has been proposed. This method creates a discrete without-replacement distribution using the marginals and uses quasirandom sampling to draw the individuals. It guarantees that the randomly sampled population will exactly match the marginal data without integerisation step needed. Finally, a hybrid approach called Quasirandom Integer Sampling of IPF (QISI) combines IPF and QIS by constructing a distribution with IPF and then sampling the integral population without replacement. This approach provides a bridge between IPF and combinatorial optimisation, offering a compromise between the efficiency and accuracy of both techniques^42^.

Similarly, the population projection to future years may be done in various ways:

- A *dynamic projection*^23,43^ consists in adding individuals through births, removing individuals from deaths, ageing the all population, and adjusting it through migrations. However, this type of approach requires extensive knowledge about transitions between each socio-demographic attribute state if we do not want the projected individuals to be “old babies” (i.e. to age individuals without evolving their other attributes).
- A *static projection*^15^ consists in using the base synthetic population as a sample and applying a reconstruction method like IPF or QISI to make the population fit the projected marginals. However this approach may be computationally expensive and thus not usable if the number of individual’s attributes and possible attributes states in the base population are important.
- Finally, *resampling* is a simple and efficient projection approach. This consists in using the base population and randomly duplicating or removing individuals from the population in order to fit the projected marginals. This method presents the advantages to be fast, to not be data-intensive and to keep the individual attributes consistent. This is a method that we developed after noticing that 1) there was not enough information on the transitions between attributes states to apply a dynamic projection and 2) methods like QISI were not suitable if individuals had their small area of residence as an attribute, because this attribute has between 50 and 20160 possible values depending on the Province, which makes the application of the QISI method extremely slow. A limitation of the resampling method is that it assumes that the individual sets of attributes remain the same over time, which makes it more suitable for short- and medium-term forecasts, where changes in individual correlated attributes (salary by age, qualification by age, etc) are small, than for long-term forecasts. The longer-term the predictions are the more uncertainty they involve. Forecasting to 2042 may thus involve a risk if done for certain scenarios (e.g. forecasting a non-marginal change in the population structure).

This paper presents the construction and validation of a synthetic population for Canada. First, the QISI approach was used to generate a base synthetic population from the 2016 Canada census data. We used 2016 census PUMF data that are realistic at the individual level and 2016 census aggregated data that allow a geographically realistic distribution of the individuals. Then, a resampling method was used to project the base population of 2016 to present (2023) and future (2030) years based on provincial population projections. Two designed algorithms were then used to assign individuals to households and to infer household types. In addition to 2023 and 2030, a population was synthesised for 2021 and compared to population data from the 2021 census, in order to validate the approach and dataset. Comparison results are presented at the national, city and dissemination area levels, to support the technical quality of the dataset.

The 2023 and 2030 synthetic populations have been developed for the RAIM (Responsible Automation for Inclusive Mobility) project. The RAIM project is a British-Canadian collaboration to address how an on-demand autonomous vehicle system can meet the diverse needs of older populations and improve the lives of older travellers. The RAIM research is applied in two regions: the city of Winnipeg (Manitoba, Canada) and the West Midlands (UK), through partnerships with local transport providers. As part of the project, an agent-based model will be developed and simulations will be conducted to predict how demand for an on-demand autonomous vehicle service varies given spatial, temporal, and population-level variation. Such simulations require individual-level population estimates to be built for the study regions at fine spatial scale. Data produced in this paper will be used as an input for the agent-based model to identify the need for autonomous on-demand transportation in the city of Winnipeg. The 2023 and 2030 Winnipeg synthetic populations have be complemented with additional attributes (driving licence, health status) from local surveys, and will be extended with individual’s daily activity patterns to produce an activity-based model and derive the older population travel demand.

The synthetic population has been generated using only publicly available data and open-source code to ease replicability. The synthetic populations are provided as csv files for 2016 (base population), 2021 (validation population), 2023 (present population) and 2030 (future population). Synthetic populations for 2021, 2023 and 2030 are provided for 9 population growth scenarios. Moreover, the code used to generate the synthetic populations is also available together with the code that was employed for the validation and scripts to parallelize the code execution on a server.

Users can extract populations from the dataset for specific zones of interest (province, city, neighborhood) or for specific sub-populations to gain insight into relationships at a given spatial scale or for a given group. The synthetic population can be used as an input into agent-based models to investigate the potential impact of local public policies on present and future populations. The synthetic population can also be used to initialize an agent-based social simulation and study emergent phenomena that may result from local interactions. Users can enrich and extend the synthetic population dataset by linking it to other datasets. They can use their own data or public data, such as local surveys data, to add new characteristics to synthetic individuals. Users can link the synthetic population to OpenStreetMap data to add residential buildings to the households for example. If users are interested in a year other than 2023 and 2030, they can use the proposed scripts to project the 2016 synthetic population for years up to 2042 (latest date for which population projections are available for the provinces and territories). Once the 2021 census data is fully released, it will be possible to simply replace the input files from 2016 census and generate a 2021 base population which might be projected in the following years to obtain more accurate future populations.

## Methods

### Zoning system

The synthetic population generation uses the multi-level spatial zoning system defined by Statistics Canada^44^. On the top level, the study area comprises the whole Canada, which is divided in 10 provinces and 3 territories. Each province or territory is divided into census subdivisions (CSD), which is the general term for municipalities or areas treated as municipal equivalents for statistical purposes. All CSD are further divided into dissemination areas (DA), small geographic units each with an average population of 400 to 700 persons based on data from the previous census. Each DA is further divided into dissemination blocks (DB), but only census population and dwelling count data are available at this scale. DA are the smallest standard geographic areas for which all census data is disseminated. The synthetic individuals are produced for the whole Canada and are localised at the DA scale.

### Inputs

Two publicly available data sources, outlined in Table 1 are used as input: 2016 census data and 2018 population projections. Tables 2–6 show example extracts of the input files.

**Table 1:** Input data and sources.

Input	Format	Source
2016 Individual PUMF^45^	Microdata in Stata .dta format	Individuals File, 2016 Census of Population – Statistics Canada Catalogue no. 98M0001X (https://www150.statcan.gc.ca/n1/en/catalogue/98M0001X also available at https://abacus.library.ubc.ca/dataset.xhtml?persistentId=hdl:11272.1/AB2/GDJRT8)
2016 Hierarchical PUMF^46^	Microdata in Stata .dta format	Hierarchical File, 2016 Census of Population – Statistics Canada Catalogue no. 98M0002X (https://www150.statcan.gc.ca/n1/en/catalogue/98M0002X also available at https://abacus.library.ubc.ca/dataset.xhtml?persistentId=hdl:11272.1/AB2/PYYXXR)
2016 Census Profile by region^47^	Aggregate counts in .csv format	Census Profile for Canada, provinces, territories, CDs, CSDs and DAs - *REGION* only, 2016 Census – Statistics Canada Catalogue no. 98-401-X2016044 (https://www150.statcan.gc.ca/n1/en/catalogue/98-316-X2016001)
2016 Geographic Attribute File^48^	Geographic hierarchy in .csv format	Geographic Attribute File, 2016 Census – Statistics Canada Catalogue no. 92-151-2016001 (https://www150.statcan.gc.ca/n1/en/catalogue/92-151-X2016001)
2018 Population projections^49^	Projected population values in .csv format	Projected population, by projection scenario, age and sex, as of July 1 (x 1,000) – Statistics Canada Table 17–10-0057-01 (https://www150.statcan.gc.ca/t1/tbl1/en/tv.action?pid=1710005701)

**Table 2:** Extract from the 2016 Individual PUMF records.

Id.	Weight	Age group	Highest degree	Household size	Labour force status	Province	Household primary maintainer	Sex	Total income
871649	37.037277	40 to 44	Program > 2 years	4 persons	Employed - Worked in reference week	35	No	Female	6,000 $
591795	37.037277	20 to 24	Secondary school diploma or equivalent	4 persons	Employed - Worked in reference week	35	No	Male	24,000 $
838385	37.037277	40 to 44	Bachelor’s degree	4 persons	Not in the labour force - Last worked before 2015	35	No	Female	2,000 $

**Table 3:** Extract from the 2016 Hierarchical PUMF records.

Household Id.	Id.	Weight	Age group	Province	Household primary maintainer	Sex
6	61102	100.196885	0 to 9	24	No	Male
6	61103	100.196885	0 to 9	24	No	Male
7	71101	100.384035	20 to 24	35	Yes	Male

**Table 4:** Extract from the 2016 Census Profile for a dissemination area.

Characteristics	Total	Male	Female
Population, 2016	1,278		
Private dwellings occupied by usual residents	440		
Total - Age groups and average age of the population - 100% data	1280	560	715
0 to 4 years	90	40	45
5 to 9 years	90	45	50
10 to 14 years	100	45	50
15 to 19 years	85	40	45
20 to 24 years	95	50	50
25 to 29 years	75	25	50
30 to 34 years	95	45	50
35 to 39 years	90	40	50
40 to 44 years	105	45	60
45 to 49 years	105	40	60
50 to 54 years	100	45	55
55 to 59 years	60	25	35
60 to 64 years	50	15	35
65 to 69 years	50	20	30
70 to 74 years	35	15	25
75 to 79 years	25	10	10
80 to 84 years	10	5	10
85 years and over	5	0	5
Total - Private households by household size - 100% data	440		
1 person	85		
2 persons	120		
3 persons	85		
4 persons	85		
5 or more persons	70		
Total - Total income groups in 2015 for the population aged 15 years and over in private households - 100% data	1,000	430	565
Under $10,000 (including loss)	140	65	75
$10,000 to $19,999	190	65	125
$20,000 to $29,999	100	30	70
$30,000 to $39,999	95	35	60
$40,000 to $49,999	90	30	65
$50,000 to $59,999	70	40	35
$60,000 to $69,999	60	25	35
$70,000 to $79,999	45	15	25
$80,000 to $89,999	45	25	20
$90,000 to $99,999	30	20	15
$100,000 and over	90	55	30
Total - Highest certificate, diploma or degree for the population aged 15 years and over in private households - 25% sample data	1,015	440	580
No certificate, diploma or degree	170	90	80
Secondary (high) school diploma or equivalency certificate	305	110	200
Postsecondary certificate, diploma or degree	540	240	300
Total - Population aged 15 years and over by Labour force status - 25% sample data	1,020	440	575
In the labour force	745	345	400
Employed	690	320	370
Unemployed	55	25	35
Not in the labour force	270	100	170

**Table 5:** Extract from the 2016 Geographic Attribute File.

DBuid	DAuid	PRuid	PRename	CSDuid	CSDname
10020117004	10020117	10	Newfoundland and Labrador	1002006	Division No. 2, Subd. F
10020117003	10020117	10	Newfoundland and Labrador	1002006	Division No. 2, Subd. F
47090190048	47090190	47	Saskatchewan	4709006	Wallace No. 243

**Table 6:** Extract from the 2018 Population projections file.

Ref_date	Geo	DGUID	Projection scenario	Sex	Age group	Value
2023	Manitoba	2016A000246	Projection scenario LG: low-growth	Females	0 to 4 years	40900
2023	Manitoba	2016A000246	Projection scenario LG: low-growth	Females	5 to 9 years	43900
2023	Manitoba	2016A000246	Projection scenario LG: low-growth	Females	95 to 99 years	2200
2023	Manitoba	2016A000246	Projection scenario LG: low-growth	Females	100 years and over	500

#### 2016 Census data

The 2016 census data were released in various ways. For this work, we used 4 outputs from the 2016 census:

- The Individual PUMF^45^. This microdata file provides access to non-aggregated data on the characteristics of the individuals in the Canadian population. The file contains a 2.7% sample of the Canadian population and provides access to 930,421 anonymised individual records from the 2016 Census questionnaire. Each individual in this sample presents 123 variables, a unique identifier and an individual weighting factor. Individuals in the PUMF are localised at the provinces (and a group gathering the three territories) level to preserve confidentiality.
- The Hierarchical PUMF^46^. Similarly to the individual PUMF, this file provides access to non-aggregated data for a sample of 1% of the Canadian households. The file contains 343,330 individuals records related to 140,705 households, and thus enables the study of individuals in relation to their households. Each individual record is restricted to the provinces level and consists in 95 variables, a unique identifier, a household identifier and an individual weighting factor.
- The Census Profile^47^. This file contains aggregate population counts for various variables (age, sex, education, households, income, etc) and for various levels of geography, including provinces and territories, CSD and DA. We used the census profile with counts disseminated at the DA level. We used as input the census profile split into six files region by region, in order to avoid loading a 5 Gb file at once.
- The Geographic Attribute File^48^. The file contains information at the DB level, based on 2016 Census standard geographic areas with correspondences from DB to higher levels. The file is thus useful for obtaining the complete geographic hierarchy of areas with the codes and names used for each level of the geographic hierarchy. For example, the codes for all DAs belonging to a CSD or a province can be obtained from this file.

It should be noted that the PUMF files do not include people living in institutions or collective dwellings such as hospitals, nursing homes, penitentiaries or student residences. These people are estimated to represent 1.9% of the Canadian population according to 2016 Census, more than half of them living in nursing homes or residences for senior citizens. People living in collective dwellings are counted in the synthetic population but are assigned into private households and have attributes from the PUMF, i.e. attributes from people not living in collective dwellings. If the dataset is used to study people living in collective dwellings, it might therefore be necessary to adapt the synthetic population, especially when generating the households.

Moreover, to protect the confidentiality of individuals, areas with a population of less than 40 persons are not present in the census profile data and census profile counts are randomly rounded either up or down to a multiple of ‘5’ or ‘10.’

#### 2018 Population projections

The second data source is population projections for provinces and territories^49^. The national statistical agency of Canada develops population projections by age and sex every 5 years for provinces and territories, based on various assumptions on the population growth. The last projections were developed in 2018, for 2018 to 2043. The population projections gives a perspective of the future Canadian population demography according to nine scenarios. Each scenario is built on assumptions about the main components of population growth (fertility, life expectancy at birth, interprovincial migration, immigration and emigration). Five medium-growth scenarios (M1, M2, M3, M4 and M5) reflect different internal migration patterns observed in the past, low-growth (LG) and high-growth (HG) scenarios explore either lower or higher population growth than in the medium-growth scenarios, and fast-aging (FA) and slow-aging (SA) scenarios consider either faster or slower population aging than in the medium-growth scenarios.

We generated a synthetic population for each projection scenario to ensure that the model can be applied to all possible use cases. For the dataset validation we used the LG scenario, which is based on the following assumptions: the fertility rate reaches 1.4 children per woman in 2042/2043; life expectancy at birth reaches 82.6 years for males and 86.6 years for females in 2042/2043; interprovincial migration is based on linear interpolation of recently observed migration rates to rates observed over a long period of time reached in 2030/2031, and rates that remain constant thereafter; the immigration rate reaches 0.65% in 2042/2043; the annual number of non-permanent residents reaches 1,259,300 in 2043; the net emigration rate reaches 0.17% in 2042/2043.

All the input data sources used to generate the synthetic population are publicly accessible through Statistics Canada Catalogue and can be downloaded from the sources listed in Table 1. PUMF are published under the Statistics Canada Open Licence since October 2018. They can be ordered for free from Statistics Canada Catalogue^45,46^ or can be downloaded from Abacus^50,51^, a repository of open data hosted by UBC Library. The input .csv file for the population projections can be downloaded through the Statistics Canada Catalogue by selecting “Download options” and then “CSV - Download entire table “Projected population, by projection scenario, age and sex, as of July 1”.

### Workflow

**Fig. 1:**
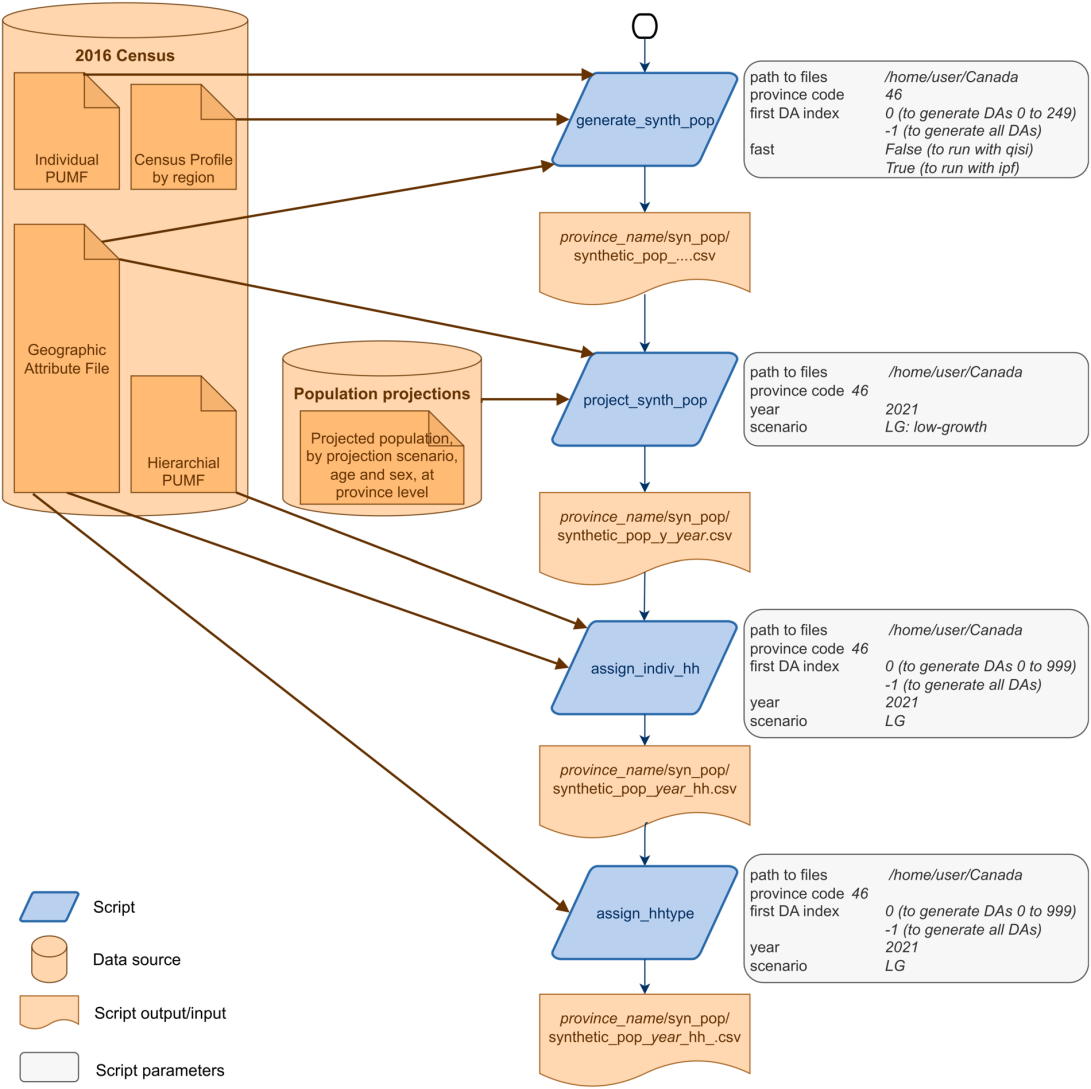
4-step workflow for generating the synthetic population. Each of the 4 scripts (in blue) takes as input (in orange) files from the 2016 census, from the population projections and an output from the previous script, as well as some parameters (in grey).

The overall workflow for generating the synthetic populations in this study is detailed in Fig. 1. The population synthesis is composed of four sequential steps: (1) generation of a base synthetic population of individuals for 2016, (2) projection of the base synthetic population towards future years 2021, 2023 and 2030, (3) assignment of individuals into households and (4) assignment of households types. On Fig. 1, scripts for each step are in blue and in orange is shown external data sources and input/output data for each script. On the right of each script, script parameters and one example of parameters are given. Each workflow step is described as follows.

#### Base synthetic population generation

The first step involves synthesising a population province by province for the base year 2016, at the DA level. The QISI approach, which combines IPF and QIS is used to synthesise an integral population DA by DA. Population synthesis for one province is performed as described in Algorithm 1.

##### Algorithm 1

Population synthesis algorithm

##### Seed initialisation

The weighted individuals localised in the province from the 2016 Individual PUMF are used to initialise the seed. Because convergence problems can occur when one of the rows is zero and the marginal total is nonzero, we allowed the zero state in the seed to be occupied with a small probability. The individuals’ variables in the seed are: age group, sex, highest degree, labour force status, household size, total income and household responsibility.

##### Marginals initialisation

The aggregate counts by DA for each variable are loaded from the 2016 Census Profile and are used as marginals (i.e. target totals) in the IPF procedure. Sometimes the subtotal for a variable is not available at the DA level. Then the distribution of the variable at the province level is used to infer the DA subtotal.

The marginals loaded for each DA are: total population, total number of households, total population by sex, total population by age group, total population by age group and sex, total population by household size, total population by highest degree, total population by labour force status, total population by income group.

The Individual PUMF variables’ categories and the Census Profile variables’ categories do not always match; e.g. categories for age group in PUMF comprise “5 to 6 years” and “7 to 9 years” while Census Profile report counts for “5 to 9 years”. We then used unified variables categories. The correspondence between categories used in the Individual PUMF, in the Census Profile, and in the synthetic population is detailed in Tables 7–13.

**Table 7:** Correspondence of the categories for the “age group” attribute.

	Code in Individual PUMF	Description in Individual PUMF	Code in synthetic population	Category in synthetic population	Characteristic in Census profile
Age group	1	0–4	0	0–4	0 to 4 years: Total
	2	5–6	1	5–9	5 to 9 years: Total
	3	7–9			
	4	10–11	2	10–14	10 to 14 years: Total
	5	12–14			
	6	15–17	3	15–19	15 to 19 years: Total
	7	18–19			
	8	20–24	4	20–24	20 to 24 years: Total
	9	25–29	5	25–29	25 to 29 years: Total
	10	30–34	6	30–34	30 to 34 years: Total
	11	35–39	7	35–39	35 to 39 years: Total
	12	40–44	8	40–44	40 to 44 years: Total
	13	45–49	9	45–49	45 to 49 years: Total
	14	50–54	10	50–54	50 to 54 years: Total
	15	55–59	11	55–59	55 to 59 years: Total
	16	60–64	12	60–64	60 to 64 years: Total
	17	65–69	13	65–69	65 to 69 years: Total
	18	70–74	14	70–74	70 to 74 years: Total
	19	75–79	15	75–79	75 to 79 years: Total
	20	80–84	16	80–84	80 to 84 years: Total
	21	> = 85	17	> = 85	85 years and over: Total
	88	Not available	ignored

**Table 8:** Correspondence of the categories for the “sex” attribute.

	Code in Individual PUMF	Description in Individual PUMF	Code in synthetic population	Category in synthetic population	Characteristic in Census profile
Sex	1	Female	0	Female	Population, 2016: Female
	2	Male	1	Male	Population, 2016: Male

**Table 9:** Correspondence of the categories for the “highest degree” attribute.

	Code in Individual PUMF	Description in Individual PUMF	Code in synthetic population	Category in synthetic population	Characteristic in Census profile
Highest degree	88	Not available	0	No certificate, diploma or degree	No certificate, diploma or degree: Total +0 to 14 years: Total
	99	Not applicable (<15 y/o)			
	1	No certificate, diploma or degree			
	2	Secondary (high) school diploma or equivalency certificate	1	Secondary school or equivalent degree	Secondary (high) school diploma or equivalency certificate: Total
	3	Trades certificate or diploma other than Certificate of Apprenticeship or Certificate of Qualification	2	Postsecondary degree	Postsecondary certificate, diploma or degree: Total
	4	Certificate of Apprenticeship or Certificate of Qualification			
	5	Program of 3 months to less than 1 year			
	6	Program of 1 to 2 years			
	7	Program of more than 2 years			
	8	University certificate or diploma below bachelor level			
	9	Bachelor’s degree			
	10	University certificate or diploma above bachelor level			
	11	Degree in medicine, dentistry, veterinary medicine or optometry			
	12	Master’s degree			
	13	Earned doctorate

**Table 10:** Correspondence of the categories for the “labour force status” attribute.

	Code in Individual PUMF	Description in Individual PUMF	Code in synthetic population	Category in synthetic population	Characteristic in Census profile
Labour force status	1	Employed - Worked in reference week	0	Employed	Employed: Total
	2	Employed - Absent in reference week			
	3	Unemployed - Temporary layoff -Did not look for work	1	Unemployed	Unemployed: Total
	4	Unemployed - Temporary layoff - Looked for full-time work			
	5	Unemployed - Temporary layoff - Looked for part-time work			
	6	Unemployed - New job - Did not look for Work			
	7	Unemployed - New job - Looked for full-time Work			
	8	Unemployed - New job - Looked for part-time work			
	9	Unemployed - Looked for full-time work			
	10	Unemployed - Looked for part-time work			
	11	Not in the labour force - Last worked in 2016	2	Not in labour force	Not in labour force: Total +0 to 14 years: Total
	12	Not in the labour force - Last worked in 2015			
	13	Not in the labour force - Last worked before 2015			
	14	Not in the labour force - Never worked			
	99	Not applicable (<15 y/o)

**Table 11:** Correspondence of the categories for the “household size” attribute.

	Code in Individual PUMF	Description in Individual PUMF	Code in synthetic population	Category in synthetic population	Characteristic in Census profile
Household size	8	Not available	0	1 person	1 person: Total
	1	1 person			
	2	2 persons	1	2 persons	2 persons: Total
	3	3 persons	2	3 persons	3 persons: Total
	4	4 persons	3	4 persons	4 persons: Total
	5	5 persons	4	5 persons or more	5 or more persons: Total
	6	6 persons			
	7	7 persons or more

**Table 12:** Correspondence of the categories for the “total income” attribute.

	Code in Individual PUMF	Description in Individual PUMF	Code in synthetic population	Category in synthetic population	Characteristic in Census profile
Total income	88,888,888	Not available	ignored		
	99,999,999	Not applicable (<15 y/o)	0	<20,000 $	Total income groups Under $10,000: Total + $10,000 to $19,999: Total + 0 to 14 years: Total
	Rounded value of the amount received by the individual in 2015				
			1	20,000 $ to 59,999 $	$20,000 to $29,999 + $30,000 to $39,999 + $40,000 to $49,999 + $50,000 to $59,999
			2	60,000 $ to 99,999 $	$60,000 to $69,999 + $70,000 to $79,999 + $80,000 to $89,999 + $90,000 to $99,999
			3	≥100,000 $	$100,000 and over

**Table 13:** Correspondence of the categories for the “primary household maintainer” attribute.

	Code in Individual PUMF	Description in Individual PUMF	Code in synthetic population	Category in synthetic population	Characteristic in Census profile
Primary household maintainer	0	Person is not primary maintainer	0	Is not primary maintainer	Population, 2016: Total - Private dwellings occupied by usual residents
	1	Person is primary maintainer	1	Is primary maintainer	Private dwellings occupied by usual residents: Total

##### Marginals matching

The subtotals sum for each variable must be equal to the DA total population count in order to apply IPF. However, categories of some of the variables in the Census Profile report counts only for the population aged 15 years and over. In order to match the total population count, we added the count of population for the age group 0–14 years to the category “No certificate, diploma or degree” for the “Highest degree” variable, to the category “Not in labour force” for the “Labour force status” variable, and to the category “<$20,000” for the “Total income” variable.

Moreover, due to missing data and randomly rounded variables to preserve confidentiality, variable totals do not always match the DA total population count. Total population counts by sex, by age group, by age group and sex, by household size, by highest degree, by labour force status, and by income group have therefore been adjusted to match the total population count. The marginals matching process is done for each variable by iteratively increasing or decreasing the variable marginals following the province marginals distribution, until the variable marginals sum match to DA total population

##### Quasirandom integer sampling of IPF (QISI)

The QISI algorithm first constructs a probability distribution for individuals, constrained to the marginal sums in every dimension, using IPF. QISI then samples the integral population using Quasirandom Integer Sampling without replacement. We used the implementation from the humanleague package^42^, developed for micro-synthesising populations from marginal and seed data.

#### Population projection

Population projections published by Canada’s national statistical agency are available by age and sex for each province or territory, for each year from 2018 to 2042, and for 9 population growth scenarios. We have projected the 2016 base synthetic population for the future years 2021, 2023 and 2030, province by province, according to each scenario.

For each scenario, each province, and each projection year, we calculated the difference in population by age group and sex between 2016 and the projection year. Then, for each age group and sex, we applied a resampling, by randomly duplicating or deleting individuals from the 2016 population in that age group and sex group to match the population of the projection year. Algorithm 2 details this approach.

##### Algorithm 2

Population projection algorithm

#### Household assignment

The third step consists in assigning the synthetic individuals into households. This step is performed for each scenario, each year of projection and each province or territory, according to Algorithm 3. At this step, an age attribute is added to each synthetic individual when the synthetic population is loaded. The age attribute is randomly drawn in the age group range of the individual. For the individuals aged 0 to 84, a uniform distribution over the age group range is used. For the individuals aged 85 and over, a geometric distribution over the age group range with a success probability p = 0.2 is used, to reflect the population rapid decline in this age group.

##### Households initialisation

For each DA, we know the number of households that need to be assigned by the number of synthetic individuals who are identified as primary household maintainer. For each DA, we then create one household by individual identified as primary household maintainer.

##### Households size determination

Then, for each household, we get the household size from the primary maintainer attributes in order to know how many members need to be assigned to this household. If the household is one person, then the household only contains the primary maintainer and is complete. If the household is more than one person, then it needs to be completed with non-responsible individuals.

##### Households completion

Each household is completed with non-responsible individuals. The non-responsible individuals are grouped by household size attribute, so that they are assigned to a household with a corresponding size. The non-responsible individuals are classified by age group either as young (age <19 years) or as adult. Young individuals are assigned into households as a priority, to avoid ending up with a high (and so unrealistic) number of young individuals not assigned to any household.

The distribution of non-responsible individuals’ age group and sex by primary maintainer’s age group and sex is inferred from the Hierarchical PUMF, for each household size. A non-responsible individual is linked to an household by randomly sampling one individual among the non-responsible individuals, according to the distribution defined by census micro-data. For example, a 2-persons household with a primary maintainer male aged 80–84 is more likely to include a female aged 80 than a female aged 0–4. This allows to preserve the distribution of household structures from the 2016 Census. If household structure is key information for the considered use case, the assignment process should be further refined. It could take into account the occupational status, education and income of individuals when assigning them into households, and include shared flats and elderly residences, for a more exhaustive representation of household relationships.

When an individual is added to an household, his HID attribute gets equal to the household identifier and the individual is removed from the pool of unassigned individuals.

##### Remaining individuals assignment

Big households (5 persons or more) in the DA are then completed with non-responsible individuals who need to be in big households and who were not assigned in the previous step. Finally, households that are not full are filled in with unassigned non-responsible individuals according to the distribution defined by census microdata. After the household assignment process, each individual has an additional age attribute and a HID attribute related to his household. In some DA, a small number of households will not be full or a small number of individuals will not be assigned to an household (because the households number and sizes do not exactly match the individuals count). The unassigned individuals have an HID attribute equal to −1.

##### Algorithm 3

Household assignment algorithm

#### Household type assignment

A final step consists in assigning a type to each household. The household type is inferred from the number of members in the household and from their age. This step is performed for each scenario, each projection year and for each province or territory.

##### Households census categorisation

Statistics Canada classifies households into 9 types: One-census-family household without additional persons: Couple without children/Couple with children/Lone parent family, One-census-family household with additional persons: Couple without children/Couple with children/ Lone parent family, Multiple-census-family household, Non-census-family households: One person household/Two or more person non-census-family household. A census family is defined as a married couple, a common-law couple or a lone parent with at least one child living in the same dwelling. Census family households contain at least one census family. Non-census-family households are either one person living alone or at least two persons who live together but do not constitute a census family.

##### Households simplified categorisation

We defined the following simplified categories for the household type: “One-person household”, “Couples without children”, “Couples with children”, “One-parent-family” and “Other kind of household”. We assigned the four most classical household types (83% of individuals in the 2016 census): “One-person household”, “Couples without children”, “Couples with children”, and “One-parent-family”, following simplistic rules regarding individuals’ ages. Other household structures (shared accommodation, more complex family household, …) are considered as “Other kind of household”. This process is simplistic in the way that it does not take into account couples with a large age difference, step families with little age difference between an adult and one of the children, or individuals living in a household without a family relationship.

##### Household type assignment process

Algorithm 4 describes the assignment process. Households composed of one individual are one-person households. Households composed of two members having more than 16 years difference are assumed to be one-parent family households. Otherwise, if both members are aged more than 16, the household is presumed to be a couple without children. For households with 3 to 6 members, the following assumptions are applied. If the two oldest members are aged more than 16 and other members are less than 16, or if the two oldest members have more than 16 years difference with the last member, the household is a couple with children. Otherwise, if the oldest member has more than 16 years difference with other members, who are all less than 16, then the household is a one-parent family. All unassigned households after this process are considered to be other kind of households.

##### Algorithm 4

Household type assignment algorithm

## Data Records

The synthetic population dataset for all Canada is public and freely available on Zenodo^52^. The dataset is composed of 364 files, organised into 13 folders, one by province or territory. Each folder is named after the province or territory and contains the synthetic population at the DA level for the province (or territory) in .csv files. The synthetic population is available for the year 2016, and for each of the nine projection scenarios for the years 2021, 2023 and 2030. The CSV files’ names refer to the year for which the synthetic population is generated. For example the file *manitoba/syn_pop/FA/synthetic_pop_2023_hh_.csv* contains the synthetic population for Manitoba for the year 2023 projected according to the fast-aging scenario (after the household assignment and household type assignment). Each csv file contains one line per individual in the following format: *index, HID, sex, prihm, agegrp, age, area, hdgree, lfact, hhsize, totinc, hhtype*. The descriptions, codes and categories of individuals attributes in the synthetic population file are listed in Table 14.

**Table 14:** Individual’s attributes in the synthetic population with their definitions and possible categories.

Variable	Definition	Categories
index	Individual identifier	Integer unique for the province
HID	Household identifier	Integer unique for the province
		-1: not assigned to an household
sex	Sex	0: female
		1: male
prihm	First person in the household identified as a household maintainer	0: not primary maintainer
		1: primary maintainer
agegrp	Age group	0: 0 to 4 years
		1: 5 to 9 years
		2: 10 to 14 years
		3: 15 to 19 years
		4: 20 to 24 years
		5: 25 to 29 years
		6: 30 to 34 years
		7: 35 to 39 years
		8: 40 to 44 years
		9: 45 to 49 years
		10: 50 to 54 years
		11: 55 to 59 years
		12: 60 to 64 years
		13: 65 to 69 years
		14: 70 to 74 years
		15: 75 to 79 years
		16: 80 to 84 years
		17: 85 years and over
age	Age in completed years	Integer ∈[0;120]
area	Dissemination area code	a 8-digit code: a 2-digit province code, followed by a 2-digit census division code, followed by a 4-digit area code.
hdgree	Highest certificate, diploma or degree	0: no certificate, diploma or degree
		1: secondary school or equivalent level
		2: postsecondary degree
lfact	Labour force status	0: employed
		1: unemployed
		2: not in labour force
hhsize	Number of individuals in the household	0: 1 person
		1: 2 persons
		2: 3 persons
		3: 4 persons
		4: 5 persons or more
totinc	Total income, receipts that tend to be of a regular and recurring nature, before income taxes and deductions	0: < 20,000 $
		1: 20,000 $ to 59,999 $
		2: 60,000 $ to 99,999 $
		3: ≥100,000 $
hhtype	Type of relation between household members	0: Couples without children
		1: Couples with children
		2: One-parent-family
		3: One-person
		4: Other kind of household

## Technical Validation

In order to assess the reliability of the method and the synthetic dataset, we generated a synthetic population for 2021 and compared its characteristics to the characteristics of the actual 2021 population as reported by the 2021 census^53^. The comparison was performed at several resolution levels: dissemination area, national and city levels. The results are presented at the city level for three cities of different sizes to illustrate the approach reliability: Toronto (most populated city in Canada, 2.8 million inhabitants), Winnipeg (6th most populated city, 749 thousand inhabitants) and Sherbrooke (30th most populated city, 173 thousand inhabitants)^54^.

At each resolution level, the population was evaluated on the 2021 census characteristics published at the time of writing, i.e.: population count, population count in private dwellings, population count by sex, population count by age range, population count by income range, households count, household count by size, and household count by type. Characteristics relative to education and labour have not been published by the national statistical agency for Canada at the time of writing and have therefore not been included in the evaluation.

There is no consensus on the appropriate validation metrics for synthetic population^14^. Following recommendations from Lovelace and Dumont^55^, validation at the DA level was performed by calculating three commonly-used metrics: Pearson’s correlation coefficient (r), Normalised Standardised Root Mean Square Error (NRMSE) and Relative Absolute Error (RAE). The metrics are defined as follows:

1 $$r=\frac{{\sum }_{i=1}^{n}\left(ob{s}_{i}-\overline{obs}\right)\left(si{m}_{i}-\overline{sim}\right)}{\sqrt{{\sum }_{i=1}^{n}{\left(ob{s}_{i}-\overline{obs}\right)}^{2}{\left(si{m}_{i}-\overline{sim}\right)}^{2}}}$$

2 $$NRMSE=\frac{\sqrt{\frac{1}{n}{\sum }_{i}^{n}{\left(ob{s}_{i}-si{m}_{i}\right)}^{2}}}{max\left(obs\right)-min\left(obs\right)}$$

3 $$RA{E}_{i}=\frac{| ob{s}_{i}-si{m}_{i}| }{ob{s}_{i}}\quad \forall \,i\in [1,\,n]$$

with *n* the total number of DAs, (*obs*_1_, *obs*_2_, ..., *obs*_n-1_, *obs*_n_) the observed counts for the attribute category under consideration and (*sim*_1_, *sim*_2_, ..., *sim*_*n*-1_, *sim*_n_) the synthetic population counts for the attribute category.

In addition to comparing the aggregated socio-demographic characteristics, we checked that the synthetic individuals were realistic. To do this, we calculated the proportion of synthetic individuals whose attribute set exactly matches one of the individuals from the 2016 census micro-data.

### Dissemination area level evaluation

The validation metrics for each attribute category across all DAs are summarised in Table 15. The metrics indicate a good fit between the synthetic population and the census population: the correlation is high (r > 0.9), the NRMSE is low (<1%) and the RAE is low (≤50% for 75% of the DAs) for almost all categories. The RAE suggest that half of the DAs represent the observed population count within a difference ≤9%, and 75% of the DAs represent the observed population count within a difference ≤14.55%. Synthetic population at the DA level is less reliable under important land-use change between censuses. Areas with very high RAE regarding population counts were manually checked with Google Maps data in order to try to understand the high error. We noticed that in these DAs important land-use changes may have occurred between censuses: construction/destruction of a residential building, reallocation of a building to a different use, or DAs where the population vary a lot on the season/day. For example for DA 35204599, the 2016 census counts 797 individuals in 272 households. The synthetic population predicts 886 individuals in 313 households for 2021, which seems realistic. However, the 2021 census counts 293 individuals in 3 private dwellings. A land-use check shows that this DA is primarily student housing, which may explain the variations in counts between censuses.

**Table 15:** Validation metrics for evaluating the 2021 synthetic population by comparing the dissemination area counts with the 2021 census population in each category.

Category	Pearson’s correlation coefficient r	NRMSE %	RAE % min/q1/median/q3/max
Population	0.951	0.705	0.0/4.58/9.0/14.55/4,760.0
Population private dwellings	0.950	0.704	0.0/4.76/9.39/15.37/8,820.0
Households	0.953	0.621	0.0/5.26/9.62/14.29/10,133.33
Males	0.951	0.704	0.0/4.86/9.82/16.3/2,728.0
Females	0.949	0.719	0.0/4.19/8.74/14.87/6,095.0
0 to 4 years	0.912	0.728	0.0/12.0/26.67/50.0/6,980.0
5 to 9 years	0.916	0.834	0.0/11.11/25.0/44.0/6,580.0
10 to 14 years	0.922	0.956	0.0/11.43/25.0/43.76/5,400.0
15 to 19 years	0.919	**1.024**	0.0/11.43/25.0/46.67/4,300.0
20 to 24 years	**0.894**	**1.064**	0.0/12.0/26.67/50.0/1,680.0
25 to 29 years	0.911	0.943	0.0/12.0/25.71/47.5/4,380.0
30 to 34 years	0.918	0.757	0.0/11.43/25.0/45.0/3,300.0
35 to 39 years	0.919	0.757	0.0/11.43/24.44/44.0/1,440.0
40 to 44 years	0.922	0.813	0.0/11.11/24.0/42.86/2,260.0
45 to 49 years	0.925	**1.022**	0.0/10.0/22.5/40.0/1,250.0
50 to 54 years	0.922	0.845	0.0/10.0/22.22/40.0/830.0
55 to 59 years	0.925	0.638	0.0/10.0/20.0/36.67/1,960.0
60 to 64 years	0.925	0.534	0.0/10.0/21.11/37.5/1,240.0
65 to 69 years	0.923	0.524	0.0/10.77/22.86/40.0/940.0
70 to 74 years	0.917	0.547	0.0/12.86/26.67/46.67/940.0
75 to 79 years	**0.887**	0.680	**0.0**/**16.0**/**33.33**/**60.0**/**1,620.0**
80 to 84 years	**0.865**	0.908	**0.0**/**20.0**/**40.0**/**75.0**/**1,900.0**
85 to 89 years	**0.813**	**1.838**	**0.0**/**20.0**/**46.67**/**80.0**/**2,190.0**
90 to 94 years	**0.784**	**1.190**	**0.0**/**22.86**/**50.0**/**80.0**/**1,540.0**
95 to 99 years	**0.664**	**1.579**	**0.0**/**40.0**/**60.0**/**80.0**/**340.0**
100 years and over	**0.391**	**5.193**	**0.0**/**40.0**/**60.0**/**80.0**/**460.0**
1 person	0.935	0.862	0.0/6.67/13.85/25.33/5,760.0
2 persons	0.947	0.512	0.0/6.15/13.33/23.33/880.0
3 persons	0.936	0.822	0.0/8.89/20.0/36.0/1,100.0
4 persons	0.943	0.732	0.0/10.0/20.0/40.0/960.0
5 persons or more	0.919	0.665	**0.0**/**13.33**/**30.0**/**60.0**/**2,860.0**
<20,000 $	0.934	**1.756**	**0.0**/**54.17**/**75.71**/**100.65**/**1,620.0**
20,000 $ to 59,999 $	0.944	0.691	0.0/4.44/9.47/16.8/335.86
60,000 $ to 99,999 $	0.941	0.841	0.0/8.24/17.14/29.52/1,980.0
≥100,000 $	0.919	0.875	0.0/13.12/26.67/45.0/2,980.0
Couples without children	0.942	0.504	0.0/9.33/20.0/40.0/1,840.0
Couples with children	0.920	**1.117**	0.0/10.0/20.0/31.71/1,280.0
One-parent-family	**0.819**	**1.380**	**0.0**/**20.0**/**37.5**/**64.0**/**1,420.0**
One-person	0.935	0.861	0.0/7.0/15.0/26.67/5,760.0
Other kind of household	**0.832**	**1.086**	**0.0**/**20.0**/**47.78**/**100.0**/**4,300.0**

The Pearson’s correlation coefficient r, the Normalized Relative Mean Square Error (NRMSE) and the Relative Absolute Error (RAE) are indicated. RAE statistics for all dissemination areas are given with the minimum, first quartile, median, third quartile and maximum. The values in bold are the biggest errors.

The synthetic population at the DA level is less reliable for the categories: “75–79 years”, “80–84 years”, “85–89 years”, “90–94 years”, “95–99 years”, “100 years and over”, “Income < 20,000$”, “Household with 5 persons or more”, “One-parent-family” and “Other kind of household”.

For the “Income < 20,000$” category, this is because the individuals incomes from 2016 are kept, without taking into account the salary increase. If the dataset is used with particular interest for salaries, a qualification-based salary increase should be applied to update the individual income attribute. Similarly, for the same age, people are more qualified in 2021 than in 2016. The qualification attribute will need to be adjusted with the 2021 census data once available.

For the other categories, those representing the lowest proportions of the population are the least reliable at the DA level. According to the 2021 census: age groups over 74 years old each represent between 0.03% (for 100 years and over) and 3.4% (for 75–79 years old) of the population, households of 5 persons or more represent 8.4% of households, one-parent-families and other kind of households represent 8.7% and 11.1% of households respectively. These categories have the highest RAE. The error is partly explained by the fact that census profile counts are randomly rounded either up or down to a multiple of ‘5’. The average absolute error of any value is 2.5 but the smaller the count, the larger this error as a percentage of its value. The average relative error for a population count of 1000 is 0.25%, but if the count is 10 (as it is often the case for the low proportion categories at the DA level), the error is 25%.

Finally, at the DA level, 94.3% of the individuals are realistic on average, with 75% of DAs having more than 95.4% realistic individuals.

### National level evaluation

**Fig. 2:**
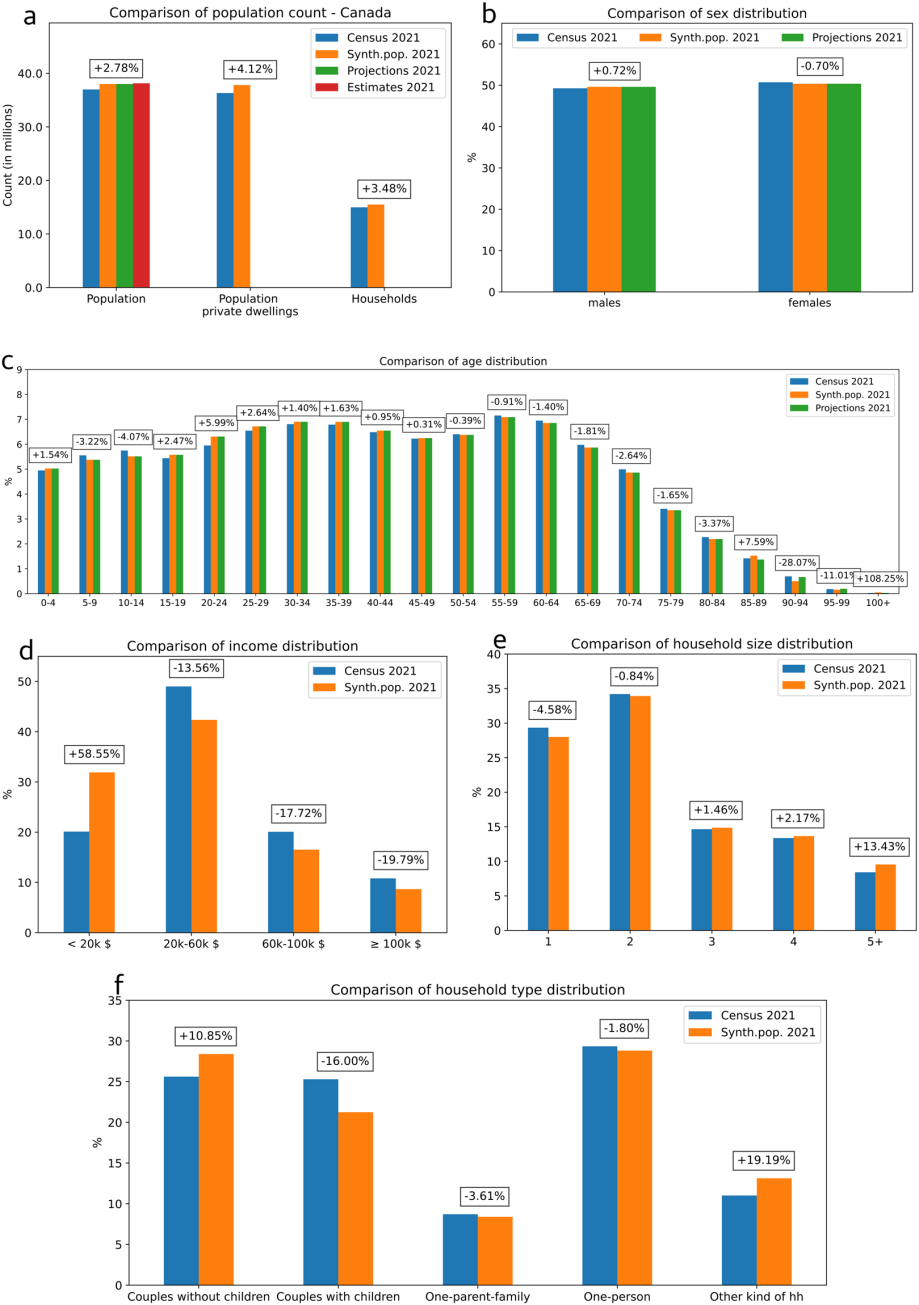
National level validation: comparison of the 2021 census population (in blue) with the 2021 synthetic population (in orange) on (**a**) the population and households counts, (**b**) the sex distribution, (**c**) the age distribution, (**d**) the income distribution, (**e**) the household size distribution and (**f**) the household type distribution. Relative errors are indicated in boxes for each category. 2021 population projections and estimates appear in green and red respectively.

Figure 2 presents a comparison of the 2021 synthetic population and the 2021 census data at the national level. Histograms show comparisons regarding the population count, population count in private dwellings, sex distribution, age distribution, income distribution, households count, household size distribution, and household type distribution. The relative error for each category appears in boxes on the histograms.

Population counts and distributions by sex and by age in the 2021 synthetic population show little difference from the 2021 census and are similar to the 2021 projections and estimates. The census population counts are not adjusted for undercoverage or overcoverage, so the population projections and estimates differ from the census and are generally higher and closer to reality. This difference is reflected in the synthetic population and accounts for part of the difference with the 2021 census. For instance, the + 5.99% error for the age range “20–24” means that while census 2021 reports 6% of population is aged 20–24 years old, our model predicted 6.36%. The 2021 synthetic population provides a good prediction of the distribution for the household types and sizes. Similarly to the DA level, the prediction is less reliable regarding the income distribution because this attribute distribution evolved from 2016 to 2021 and has not been adjusted. Finally, at the national level, 95.7% of all synthetic individuals present realistic sets of attributes.

### City level evaluation

**Fig. 3:**
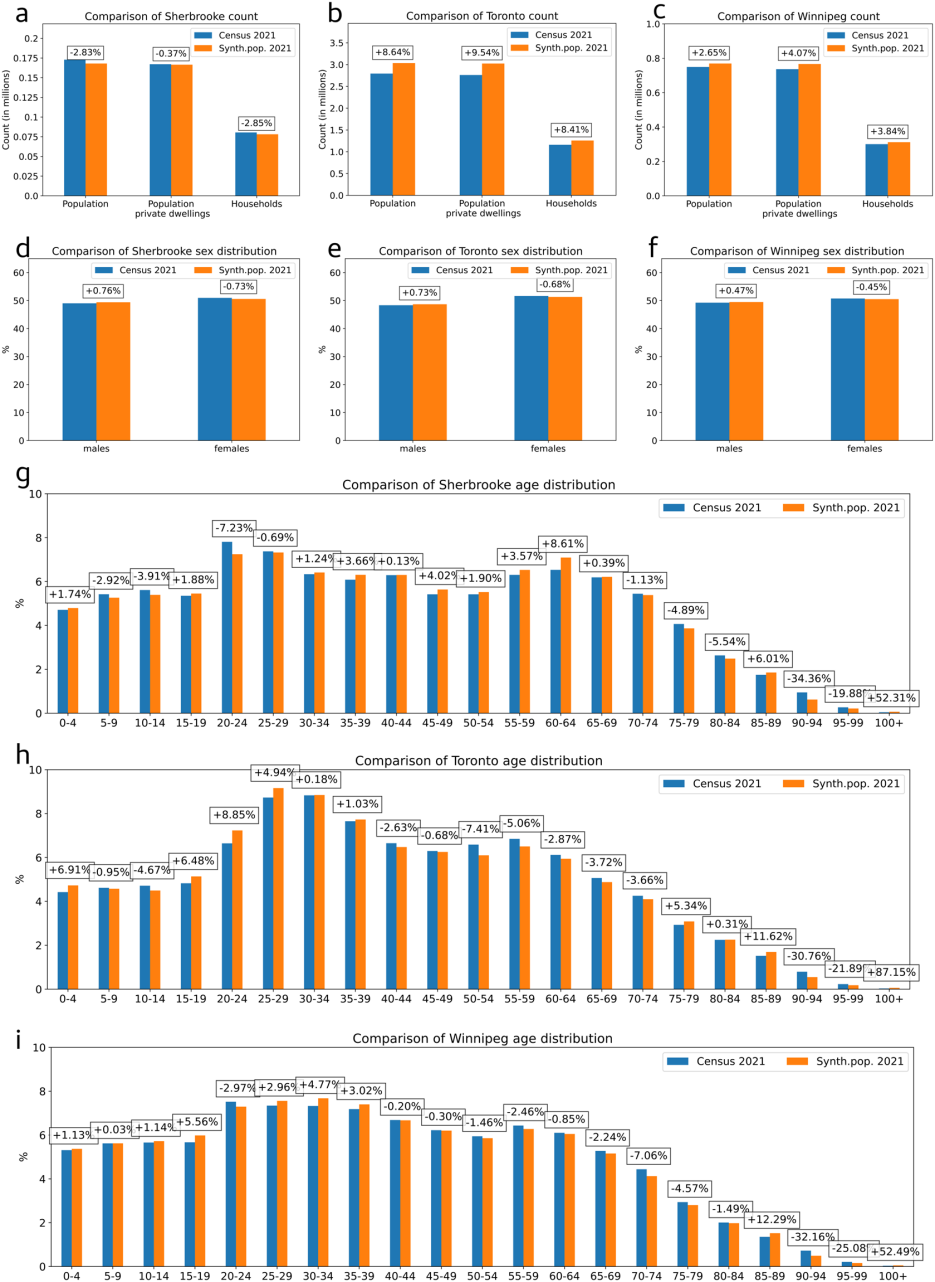
City level validation: comparison of the 2021 census population (in blue) with the 2021 synthetic population (in orange) for Sherbrooke, Toronto and Winnipeg on (**a–c**) the population and households counts, (**d–f**) the sex distribution and (**g–i**) the age distribution. Relative errors are indicated in boxes for each category.

**Fig. 4:**
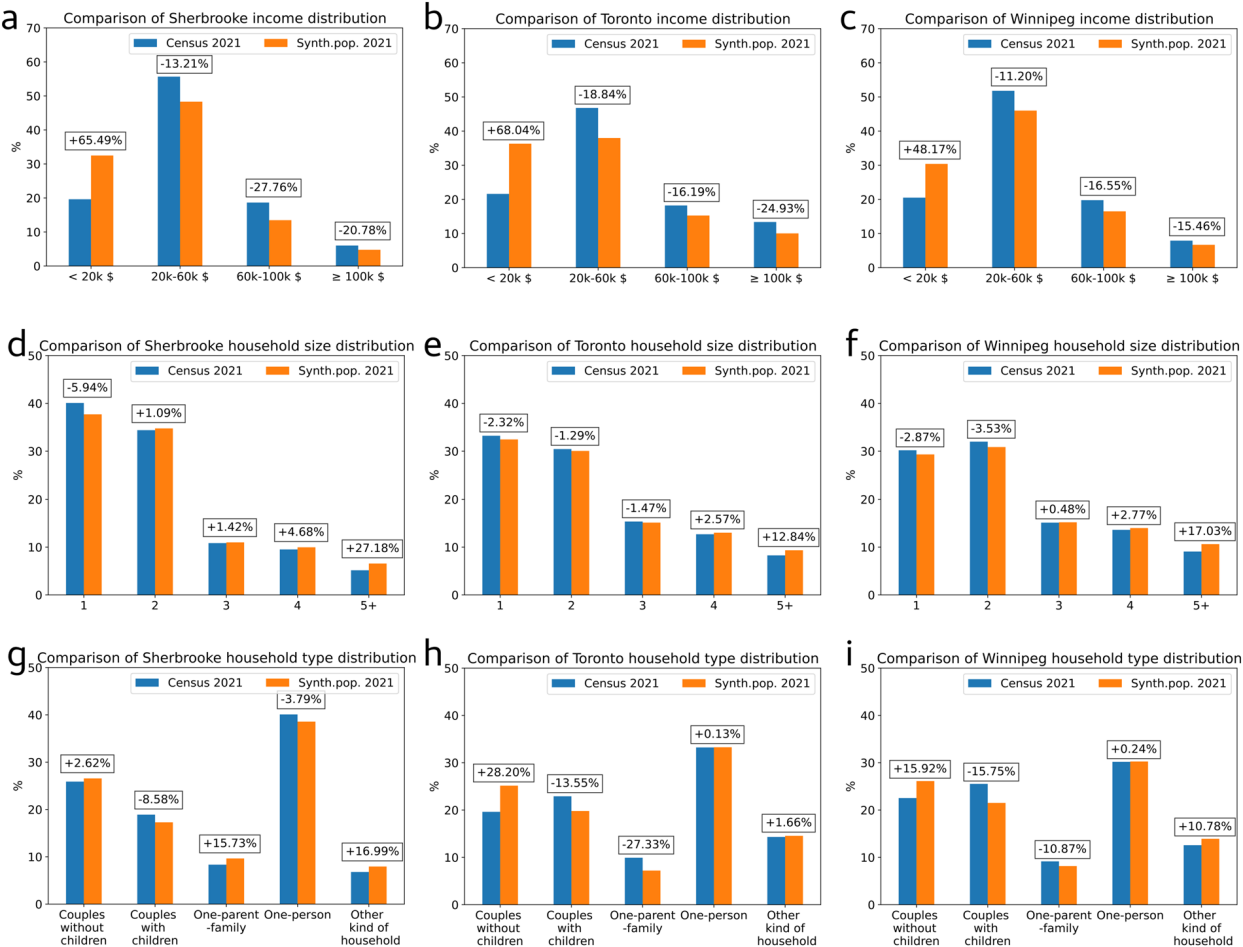
City level validation: comparison of the 2021 census population (in blue) with the 2021 synthetic population (in orange) for Sherbrooke, Toronto and Winnipeg on (**a–c**) the income distribution, (**d–f**) the household size distribution and (**g–i**) the household type distribution. Relative errors are indicated in boxes for each category.

Figures 3, 4 present a comparison of the 2021 synthetic population and the 2021 census data at the city level for Sherbrooke, Toronto and Winnipeg. Histograms and relative errors are shown for each attribute and each category.

The figures show that the 2021 synthetic population counts and distributions present a good fit with statistics from 2021 census. The synthetic population reproduces well the cities’ specificities: for example, a high proportion of 25–34 years old in Toronto and a high number of one-person households in Sherbrooke. Moreover, Sherbrooke’s synthetic population has 97.3% of realistic individuals on average (95% of DAs with >90.2% of realistic individuals), Toronto’s synthetic population has 96.3% realistic individuals on average (95% of DAs with >92.4% of realistic individuals), and Winnipeg’s synthetic population has 95.5% realistic individuals on average (95% of DAs with >89% of realistic individuals).

**Fig. 5:**
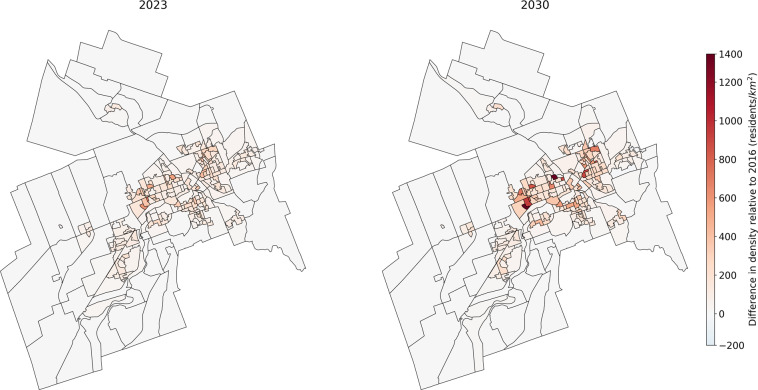
Sherbrooke synthetic population density by dissemination area for 2023 and 2030 relative to 2016.

**Fig. 6:**
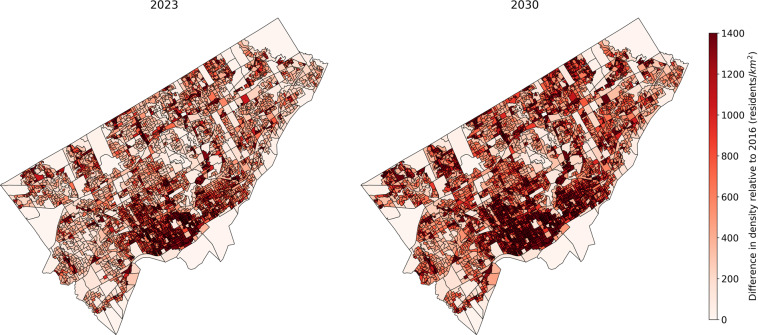
Toronto synthetic population density by dissemination area for 2023 and 2030 relative to 2016.

**Fig. 7:**
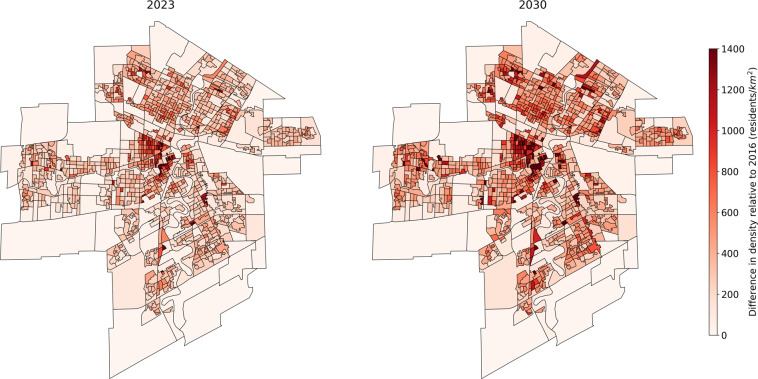
Winnipeg synthetic population density by dissemination area for 2023 and 2030 relative to 2016.

Finally, in order to illustrate the 2023 and 2030 synthetic populations, the evolution of the synthetic population density by DA from 2016 to 2023 and from 2016 to 2030 is presented for each city in Figs. 5–7. The DAs boundaries are the ones from 2016 census. A population densification can be observed in almost all areas, with greater densification in already dense areas. This is due to the way the population is projected in the future years. If the projection predicts an increase in the province population, then some synthetic individuals are drawn randomly from the 2016 province’s synthetic population to be duplicated (according to the age and sex projections) in order to expand the province synthetic population. A highly populated DA in 2016 is therefore more likely to have its individuals duplicated than a sparsely populated DA.

## Usage Notes

The synthetic population can be used directly to initialise agent-based models. Synthetic populations for specific zones can be extracted from the population dataset by identifying the zone’s geographical code in the 2016 Geographic Attribute File, getting the corresponding DA codes and filtering the synthetic individuals which have their “area” attribute within the selected DA codes. DAs boundaries can be geolocalised using the 2016 census boundaries file provided by Statistics Canada^56^. Figures 5–7 give an example of the DA spatial resolution for three cities.

For proper use of the data, it is important to note that the projected populations are independent from one year to another. This means, for example, that the individual with index 2 in the Manitoba population for 2021 is not the same as index 2 in the Manitoba population for 2023.

The generation process is partly stochastic which induces some limitations. We provide only one instance of the synthetic population by year and scenario and while the model seems stable, additional analyses of the variance between instantiations should be performed. We provide the code for users who would like to generate multiple instances of the model and perform a sensitivity analysis. In addition, if the users want to generate a synthetic population themselves (for a different projection year or using different methods of assigning households or household types), the scripts developed for this work are provided. The scripts workflow with the input files and parameters of each script are described in Fig. 1.

If the population to be synthesise covers a large area, HPC facilities are required to run the scripts in a reasonable time. The synthetic population was generated on ARC4, which is part of the High Performance Computing facilities at the University of Leeds, UK. ARC4 is a Linux-based HPC cluster, based on the CentOS 7 distribution, supporting Son of Grid Engine to run parallel batch jobs. The generation script can be parallelised to generate DAs 250 by 250 for each province. The projection script is fast and does not need to be parallelised. The household assignment and household type assignment can be parallelised to generate DAs 1,000 by 1,000 for each province. Shell scripts to run Python scripts in parallel on HPC facilities are provided with the code, as well as additional Python scripts to merge output files that were generated in parallel. The parallelisation process is documented to guide the user in its execution.

## Data Availability

The python scripts (python 3.10) developed for the generation and validation of the synthetic dataset are publicly and freely accessible on Zenodo^57^. The scripts use the following python packages: pandas (1.4.4), numpy (1.23.2), pyreadstat (1.19), scipy (1.9.1) for the Pearson’s correlation coefficient computation, scikit-learn (1.1.2) for the RMSE computation, and matplotlib (3.5.3) to generate the charts. All these python packages are available from the Python Package Index: https://pypi.org/. The humanleague package (2.1.10) providing the QISI and IPF implementations is available from the Python Package Index and on Zenodo^42^.
